# Navigating the Nonylphenol Hazard: Insights into Toxicity Mechanisms and Mitigation Approaches

**DOI:** 10.3390/toxics14100885

**Published:** 2026-10-01

**Authors:** Yonger Yu, Liang Hong, Chun Chen, Zihan Xing, Yuefei Sun, Fei Wang, Tongyu Wu, Cun Li, Shusheng Tang, Daowen Li, Xingyao Pei

**Affiliations:** 1 Tianjin Key Laboratory of Agricultural Animal Breeding and Healthy Husbandry, College of Animal Science and Veterinary Medicine, Tianjin Agricultural University, Tianjin 300384, China; 2 State Key Laboratory of Veterinary Public Health and Safety, College of Veterinary Medicine, China Agricultural University, Beijing 100193, China; 3 Technology Innovation Center for Food Safety Surveillance and Detection (Hainan), Sanya Institute of China Agricultural University, Sanya 572025, China

**Keywords:** nonylphenol, toxic mechanism, pollution profile, risk assessment, prevention strategy

## Abstract

Nonylphenol (NP), a prototypical endocrine-disrupting chemical and persistent organic pollutant, is ubiquitously detected in water, soil, and atmosphere as a degradation product of nonylphenol polyoxyethylene ethers (NPEO) widely used in industrial and consumer products. Its environmental persistence, low metabolic clearance, and high lipophilicity drive bioaccumulation in wildlife and humans, posing sustained risks to ecosystems and public health. Guided by a systematic literature search strategy across Web of Science and PubMed databases up to January 2026, this review synthesizes current knowledge on the environmental fate, toxicokinetics, multi-organ toxic effects, molecular mechanisms, and existing risk management strategies of NP. The synthesized evidence reveals that NP exposure induces systemic toxicity affecting the nervous, reproductive, endocrine, hepatic, renal, gastrointestinal, respiratory, and immune systems. Mechanistically, despite its chemical simplicity, NP converges on shared pathophysiological pathways defined by oxidative stress-driven mitochondrial dysfunction, endoplasmic reticulum stress, and programmed cell death via apoptosis, autophagy, and ferroptosis. Under environmentally realistic conditions, NP frequently coexists with microplastics, heavy metals, and other pollutants, often eliciting synergistic toxicity. Regarding risk mitigation, microbial degradation and advanced oxidation processes have demonstrated potential at the laboratory scale, while experimental studies have identified several natural compounds and trace elements as candidate protective agents. Despite substantial progress in characterizing NP’s toxicity network, significant gaps remain in isomer-specific toxicology, combined exposure assessment, and translation of remediation technologies to field applications. Future research should integrate high-resolution isomer-resolved toxicity profiling, multi-pollutant joint risk assessment models, and long-term human cohort monitoring to establish a more robust scientific framework for NP management and control.

## 1. Introduction

The advancement of society and the development of technology have brought unprecedented convenience, yet they have also engendered a series of environmental legacy issues that demand urgent scientific attention. Among these, nonylphenol (NP) stands out as a prototypical industrial legacy pollutant and a persistent ecological threat. As a ubiquitous degradation product of nonylphenol polyoxyethylene ether (NPEO) surfactants, NP possesses the dual hazardous attributes of an endocrine-disrupting chemical and a persistent organic pollutant [1,2]. Its environmental persistence, significant bioaccumulation potential and multi-effect toxicity cross the boundaries of ecosystem zones, posing a hidden risk to aquatic and terrestrial biological communities. The non-invasive biological monitoring results of Pygoscelis samples (feathers, guano and eggshells) collected on King George Island during the southern hemisphere summer of 2023/2024 showed that the NP detection rate was 76%, ranking second among the screened endocrine disruptors [3]. NP is mainly derived from industrial and domestic wastewater discharge and can be distributed in water, sediment and soil, with a special affinity for regions rich in organic matter [4,5,6,7,8]. Its semi-volatile character further promotes long-range atmospheric migration, allowing the pollution footprint to extend beyond local point sources [9].

In terms of exposure routes, livestock may ingest NP through contaminated drinking water, forage, and feed; aquatic organisms are directly exposed to NP residues in water bodies and sediments; and wildlife can encounter NP exposure at multiple trophic levels. What is more noteworthy is that NP accumulates along the food chain, resulting in detectable residues in animal-derived foods, thereby creating a direct exposure pathway for humans and exacerbating food safety concerns within the “One health” framework [4,10,11,12,13,14]. At the toxicological mechanism level, the toxic effects of NP can trigger complex cascading reactions ranging from subcellular disorders to multi-system pathologies. Based on the literature identified through our systematic search, mitochondrial dysfunction [15] and oxidative stress [16] are recognized as the main driving factors of NP toxicity kinetics. Furthermore, NP can induce endoplasmic reticulum stress [17] and disrupt the structural integrity of the Golgi apparatus [18], thereby impairing protein synthesis and cell secretion functions. These molecular-level damages occur in various cell lineages, including reproductive cells, neurons, and liver cells, ultimately leading to programmed cell death through apoptosis, autophagy dysregulation, and ferroptosis pathways [19,20,21,22]. Moreover, both clinical and subclinical outcomes in animals exhibit considerable heterogeneity and severity. The multi-system toxicity profile of NP has covered damage to reproductive [23], neurological [24], digestive [25], endocrine [26], immunological [27], respiratory [28], hepatic, and renal systems [29], and all studies have received extensive attention. For instance, NP exposure has been proven to activate Pink1/Parkin-mediated mitochondrial autophagy, promote lipid droplet degradation within hepatic stellate cells, and ultimately induce liver fibrosis [30].

In terms of analysis and detection, the current quantitative methods for NP mainly rely on the chromatography–mass spectrometry combined platform to achieve precise determination of NP and its coexisting pollutants in biological and environmental matrices. At the same time, the progress of new monitoring strategies is gradually revealing the interaction mechanisms in the context of combined exposure [31]. It is worth noting that the molecularly imprinted polymer-assisted gas chromatography–mass spectrometry (MIP–GC–MS) platform has been successfully applied for the simultaneous detection of bisphenol A and NP in river water, and this method shows significantly enhanced selectivity, minimal matrix interference, and detection limits ranging from ng·L^−1^ to ng·g^−1^ [32]. According to environmental monitoring data and experimental studies retrieved from our systematic search, NP rarely exists alone, and its coexistence with other endocrine disruptors may trigger synergistic or antagonistic toxicological effects, thereby further increasing the overall risk burden [33]. Encouragingly, a series of risk mitigation strategies have begun to emerge. Experimental model studies have shown that natural compounds such as Resveratrol (RES) and Naringenin (NG) exhibit potential in antagonizing the toxicity of NP [34]. Advanced oxidation processes [35] and microbial degradation [36] are remediation technologies that provide feasible approaches for environmental governance. Additionally, the nano-plant synergistic remediation system combining functional carbon nanodots with marigold (*Tagetes erecta* L.) can effectively remove NP from contaminated soil. Specifically, in low-concentration (5 mg/kg) and high-concentration (50 mg/kg) NP-contaminated soil, the addition of functional carbon nanodots further increased the NP removal efficiency by 55.9% and 118.3%, respectively [37]. However, how to bridge the gap between laboratory-based toxicological findings and on-site remediation technologies remains a core challenge currently faced.

### Literature Search Strategy

In the present study, we utilized core search keywords including “Nonylphenol”, and diverse types of “organ toxicity” to systematically retrieve and screen peer-reviewed studies focusing on the multi-organ toxicological effects of nonylphenol and its underlying molecular mechanisms. All eligible research literature was collected from authoritative biomedical and scientific databases, including Web of Science and PubMed, with the retrieval time frame covering all publicly available publications up to January 2026. After strict screening, classification and systematic collation of the retrieved literature, we comprehensively summarized and deeply discussed the research progress of NP-induced toxic injuries in different organs, as well as the key molecular pathways, signaling regulation and pathological mechanisms involved in NP toxicity. Moreover, this review further explores the toxicological characteristics and dose-effect relationships of NP, the internal toxic metabolism process in organisms, potential toxicological risks and research gaps in current NP toxicity studies, as well as potential intervention and mitigation strategies to alleviate NP-induced organ toxicity, along with prospective research directions for optimizing NP toxicity mitigation approaches. We hope this review can provide a comprehensive and systematic understanding of the toxic effects and molecular pathogenesis of nonylphenol and offer reliable theoretical references and valuable insights for further in-depth basic research and toxicological evaluation of NP.

## 2. Basic Characteristics of Nonylphenol

NP (C_15_H_24_O) is characterized by an alkylphenol structure consisting of a phenolic hydroxyl group linked to a long aliphatic chain. This molecular configuration confers dual properties, enabling NP to function both as an environmental estrogen and a persistent organic pollutant. NP is primarily synthesized via the alkylation of phenol with nonene. However, the alkyl substituent exhibits significant structural heterogeneity, as variations in chain length, branching, and regiochemistry result in a complex mixture of isomers.

Consequently, technical-grade NP comprises a complex mixture of more than 100 isomers and homologues [1]. Studies have demonstrated that the substitution pattern at the benzylic (α-) carbon critically determines biodegradability: isomers with quaternary benzylic carbon atoms serve as growth substrates and are preferentially degraded, with degradation rates positively correlating with the degree of branching, whereas isomers bearing benzylic hydrogens are not utilized as sole carbon sources, though they may be cometabolically transformed in mixtures [38,39]. This isomer-specific degradation behavior has direct implications for the interpretation of NP’s environmental persistence and the assessment of its ecological risks.

Empirical evidence indicates that individual NP isomers exhibit marked differences in biodegradation kinetics and estrogenic activity, resulting in distinct environmental fates and toxicological outcomes [1,2,40]. The primary precursors of NP, nonylphenol polyethoxylates (NPnEOs), constitute a widely used class of nonionic surfactants. These compounds are widely used across various industrial sectors including textile manufacturing, detergent formulation, and paper production [41,42]. Following environmental release, the ethoxylate chains of NPnEOs undergo microbial cleavage, producing estrogenic metabolites such as nonylphenol diethoxylate (NP2EO) and nonylphenol monoethoxylate (NP1EO). Subsequent metabolic pathways diverge depending on oxygen availability. Under aerobic conditions, specific pH and temperature ranges promote the formation of transformation products such as nonylphenoxyethoxyacetic acid (NP2EC) and nonylphenoxyacetic acid (NP1EC). Under anaerobic conditions, reductive conversion yields NP2EO and NP1EO. These intermediates ultimately degrade to NP, which constitutes a major source of environmental contamination [43,44].

## 3. Analytical Techniques for Nonylphenol

### 3.1. Instrumental Analytical Methods

The early developed methods for detecting NP mainly relied on instrumental analysis, featuring high precision, trace-level sensitivity, and applicability to high-throughput and complex matrix analysis. Validation studies have shown that the combination of pressurized liquid extraction (PLE) and gas chromatography–mass spectrometry (GC-MS) can provide a reliable method for the quantitative determination of trace NP residues in complex sediment samples [31]. Solid-phase extraction (SPE) combined with ultra-high performance liquid chromatography–tandem mass spectrometry (UPLC-MS/MS) is a powerful analytical platform. This method enables the detection of NP and coexisting endocrine disruptors in human urine and can also be used for NP analysis in wastewater. Its applicability is thus significantly expanded [45].

Subsequently, the GC-MS method was optimized by adding derivatization steps (using BSTFA + TMCS, 70 °C reaction for 30 min), acetone extraction, and 5 min ultrasonic treatment for the analysis of sewage and sludge samples. This optimized method successfully achieved reliable quantitative determination of NP in sludge samples from Turkish wastewater treatment plants [46].

Recent advances have enabled simultaneous quantification of NP isomers and their precursor compounds. High-performance liquid chromatography coupled with electrospray ionization mass spectrometry (HPLC–ESI–MS) enables sensitive detection of NP and NPEOs, with limits of detection as low as 1–55 pg [47]. For NP and NPEO analysis in sewage and sludge, microwave-assisted extraction (MAE) achieves a detection limit of 2.86 μg/g. This method has been successfully applied to samples from wastewater treatment plants in Athens, Patras, and Heraklion, Greece [48]. A GC–FID–MS system integrated with a Deans switch enables selective analysis and quantification of ten NP isomers in complex environmental samples [49]. An UPLC–MS/MS has been developed for NP quantification in liquid and solid wastewater matrices. This platform enables simultaneous quantification of NP and 15 precursors in aqueous and solid phases, with detection limits of 1.4–17.4 ng/L and 1.4–39.4 ng/g, respectively [50]. With advances in analytical technology, methods for concentrating NP from leachates and wastewater have been developed. These methods integrate simultaneous distillation extraction, full two-dimensional gas chromatography, and time-of-flight mass spectrometry for sample pretreatment and analysis, thereby improving the efficiency of separation and identification of NP isomers [51]. In another study, GC-MS combined with BSTFA derivatization enabled the integrated analysis of NP, NP1EO, NP2EO, bisphenol A, and triclosan (TCS) in wastewater (dissolved and particulate phases) and sewage sludge. These findings suggest that NP detection has evolved toward multiple analytical methods for environmental EDCs [52].

### 3.2. Rapid Immunoassay System

Driven by the needs of field detection and continuous monitoring, a new class of rapid immunodetection methods for NP has emerged. These systems do not require large instruments and expertise, and have the advantages of high specificity, sensitivity, low cost and rapid detection. Recent studies have developed rapid immunosensors using NP specific aptamers selected from single-stranded DNA libraries, combined with gold nanoparticles (AuNPs) [53]. Another study also developed an immunosensor for NP detection, which immobilized specific antibodies and horseradish peroxidase (HRP) on a carbon screen-printed electrode. The detection mechanism is based on the enzymatic reaction signal changes caused by NP, which come from the oxidation of the catalytic media (such as methylene blue, hydroquinone, iodide) catalyzed by HRP. This method can achieve an adjustable sensitivity of 20–44 μg/L by adjusting the nature, concentration and reaction kinetics of the medium [54].

## 4. Regulatory Policies and Control Standards for Nonylphenol

Given widespread environmental occurrence and documented toxicological effects, NP is subject to a range of regulatory controls aimed at reducing its ecological and health impacts. Under the EU REACH Regulation, a mandatory threshold limit for NP in consumer products has been established. It stipulates that products containing ≥0.1% NP shall not be placed on the market. Such products are also prohibited for use in the manufacture of textiles and cosmetics [55,56]. In the United States, some states have established advisory limits for NP in drinking water. For example, Minnesota sets a maximum concentration of 20 μg/L [57]. In Japan, NP is listed as a Hazardous Air Pollutant under the Air Pollution Control Act, requiring ongoing environmental monitoring and emission reporting [58]. China has issued the Priority Control List of New Pollutants, which outlines risk management measures for restricting and prohibiting NP. These measures include banning NP as an additive in pesticide production and as an ingredient in cosmetics. They also prohibit the use of NP in the production of NP ethoxylates (NPEOs) [59]. The accurate assessment of the risk of NP exposure relies on monitoring tools with high sensitivity and precision. The effective implementation of these regulations also depends on such tools. Although current detection technologies have limitations, there is a clear need for further development of reliable and robust quantitative methods. The summary of the regulatory measures is shown in Table 1.

## 5. Environmental Fate and Transformation of Nonylphenol

### 5.1. The Ecological Destructiveness of Nonylphenol

The evidence indicates that the NP discharged by industries has a strong pervasive diffusion and distribution effect on water bodies, soil and the atmosphere, posing threats to wildlife, livestock farming and public health [4,5,6,7]. Firstly, NP can enter the water matrix through wastewater, agricultural runoff, and soil groundwater leakage. The current pollution status of water environment has been widely investigated. Risk assessment reports have indicated the risks of NP, suggesting that NP is ubiquitous in water bodies in China. The concentration value at the sampling point in the Pearl River estuary is within the range of 233.04 to 3352.86 ng/L, which is the main detected endocrine-disrupting compound [60]. Moreover, NP in water bodies may further cause toxic damage to aquatic organisms. For example, continuous exposure to 5, 15 and 50 μg/L of NP for 3 days and 7 days is sufficient to induce disorder in estrogen secretion in the surface mucus of immature juvenile Atlantic salmon (*Salmo salar*) [61]. Surface water pollution is only one of the consequences of NP entering water bodies. More NP is detected in the sediment of water bodies [8]. Furthermore, with the intensification of global warming and the increase in glacier melting, it is easy to predict that the transportation of NP to downstream environments will also increase [62]. Due to its physical and chemical properties of low solubility and high hydrophobicity, NP is prone to accumulate in environmental compartments containing high organic matter. Therefore, in addition to causing pollution to the water environment, NP is easily adhered to the organic surfaces of soil and sludge and transferred to organisms through soil-related ecological enrichment [2,41,43]. In agricultural production, NP is often used as a plasticizer and pesticide emulsifier and can pollute the soil through various pathways such as sewage irrigation, sludge use in agriculture and pesticide application [63,64]. It is reported that the adsorption process of NP in soil includes two main stages: rapid adsorption and slow adsorption. Compared with inland soil, NP is more likely to be adsorbed on the soil in coastal areas [64,65].

**Figure 1:**
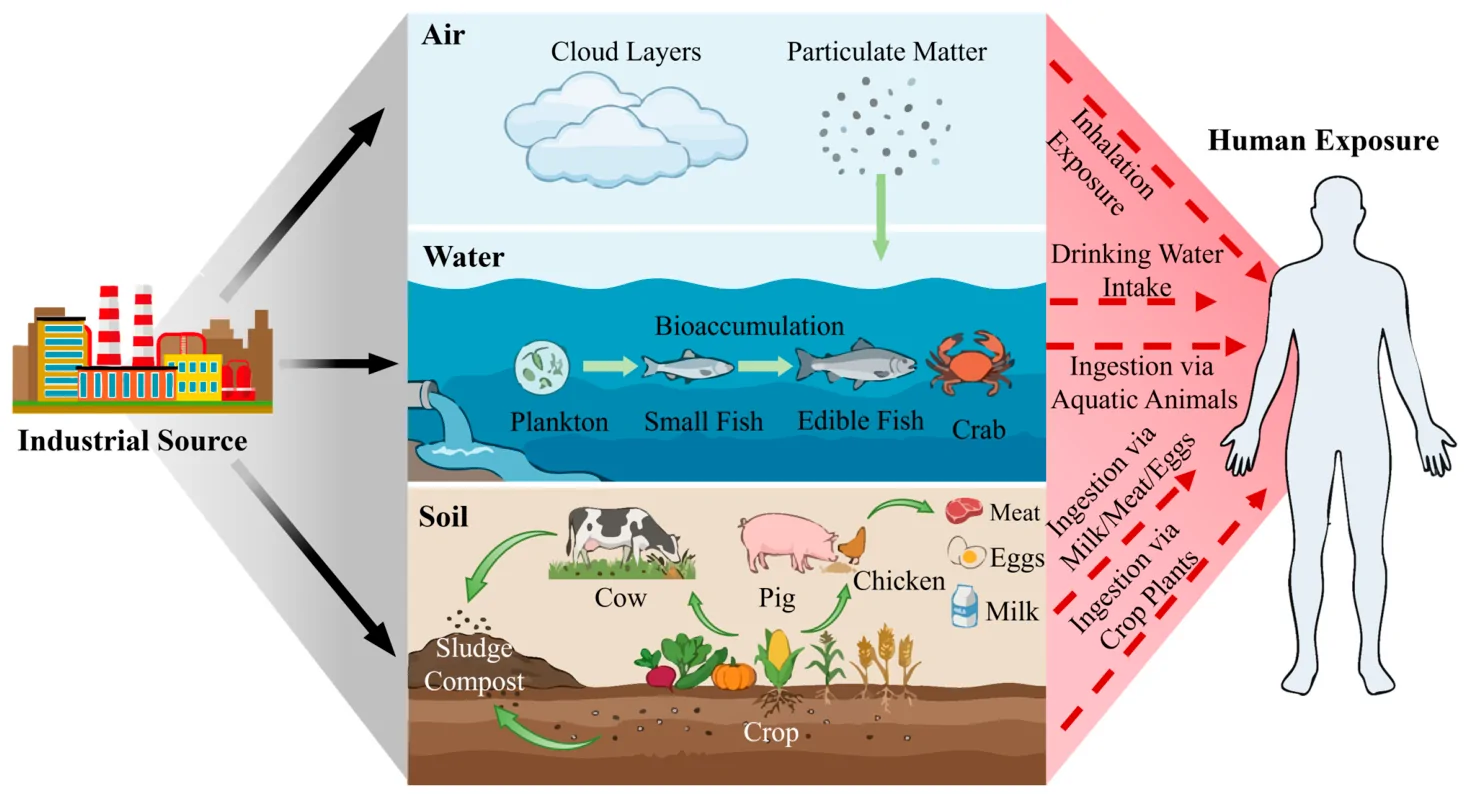
Environmental transmission and human exposure pathways of nonylphenol. The pathways and matrices depicted are based on peer-reviewed studies identified through the systematic literature search covering publications up to January 2026 (see Section 1 for search strategy).

NP exerts negative effects on plants. In mung bean (*Vigna radiata*) leaves exposed to soil containing 2000 mg/kg of NP, chlorophyll content and stomatal size were significantly reduced. These changes ultimately led to leaf necrosis. Additionally, long-term exposure of rice (*Oryza sativa*) to NP results in a significant reduction in the height of ground plants, and the formation of chlorophyll and root development functions are significantly inhibited [66]. Speculation suggests that these phenomena may be related to the persistent residual characteristics of NP. In the tested three types of soil, the initial degradation of NP is rapid (average half-life = 11.5 days), but ultimately 26% to 35% of NP is difficult to decompose. The evidence shows that high concentrations of NP residues in the soil can cause the accumulation of NP in the roots of leguminous plants, and the concentration of NP absorbed by the roots of leguminous plants is significantly higher than that of shoots and grains [67]. Air is another pollution route for NP. It has been confirmed that the atmospheric pollution caused by NP is not only from direct industrial emissions, and due to its semi-volatile property, NP from plastic products, rubber products, water bodies, and soil can also easily migrate to the atmosphere [9]. This diffusion leads to the pollution range of NP exceeding the local area where the emission source is located. The survey shows that NP can further be enriched through the food chain and threaten biological safety. NP can be detected in seafood [10,11,12], dairy products [10], meat [13], and drinking water [14] at different concentrations. More importantly, NP has been detected in human urine [45,68,69], feces [70], breast milk [10], and blood [71,72], and the risk and toxicity intervention mechanism for public health still needs to be analyzed (Figure 1).

### 5.2. Microbial Remediation Strategies for Nonylphenol

Building on the advances made in NP detection, the development of cost-effective remediation technologies is essential for source control and public health protection. Biodegradation-based approaches are one of the key strategies for NP removal within an integrated bioremediation framework. A study of four engineered algae-bacterial symbiotic systems showed that Streptomyces and XQ-3C-XQ-based symbiotic systems were the most efficient, capable of removing more than 94% of NP within 7 days. Other symbiotic systems also contribute to NP degradation and, although less active, still exhibit detectable degradation effects. These findings highlight functional redundancy and resilience in the design of microbial [36]. Defined facultative microbial consortia have demonstrated substantial NP removal capacity. At an initial concentration of 1000 mg/L, these consortia achieved 75.61% degradation within 48 h. After 8 days, degradation increased to 89.75% [73]. Similarly, NP degradation reached 83.6% within 6 days in a microbial electrolysis cell (MEC) system. This was accompanied by enrichment of key functional bacterial communities. These included Citrobacter, Desulfovibrio, and Advenella [74]. Increasing numbers of microorganisms with strong NP-degrading capabilities have been identified. White-rot fungi (WRF) produce extracellular ligninolytic enzymes. These include laccase, manganese peroxidase, lignin peroxidase, and versatile peroxidase. These enzymes collectively degrade a range of endocrine-disrupting compounds [75]. The degradation of NP (100 mg/L) by four WRF strains was evaluated. *Trametes versicolor* and *Bjerkandera* sp. BOL13 showed the highest removal, eliminating 97 mg/L and 99 mg/L NP, respectively, within 25 days [76]. The non-ligninolytic fungus *Gliocephalotrichum* simplex also demonstrated NP-degrading capability. Within 24 h, it achieved removal efficiencies of 88% and 50% at initial NP concentrations of 50 and 100 mg/L, respectively [77]. Bifidobacterium CN12 degrades NP in sewage/sludge through synergistic interactions with bacterial consortia possessing complementary metabolic pathways [78]. An increasing number of microorganisms have been identified as effective NP degraders. These include Nitrospira [79], *Pseudoxanthomonas mexicana* [80], the fungus *Umbelopsis isabellina* [81], *Metarhizium robertsii* IM 6519 [82], and sludge-isolated *Bacillus sphaericus* CT7 [83]. This diversity highlights their potential for both industrial and environmental remediation.

### 5.3. Abiotic Attenuation Strategies for Nonylphenol

In addition to microorganisms, NP degradation efficiency is influenced by the type, dosage, and conditions of organic filler. In sewage sludge, the addition of 20% mushroom residue resulted in 71.6% NP degradation, whereas 20% sawdust achieved 22.5%. Adjusting aeration time to 5–15 min increased the degradation rate by over 20% [84]. In addition to biodegradation, a pilot-scale sequencing batch reactor study showed that ozonation increased NP removal efficiency by approximately 32% [85]. Simulated solar irradiation can induce the photolysis conversion of NP to produce intermediates such as phenol, hydroquinone, and 1,4-benzoquinone. Irradiation for 80 h in the presence of hydrogen peroxide effectively reduced the estrogenic activity of NP, indicating the potential of advanced oxidation processes for environmental control [35]. The effect of temperature on the removal efficiency of NP has not been adequately studied. Decreasing the temperature from 28 °C to 10 °C in a lab-scale activated sludge system significantly reduced the biodegradation rate of NP. This finding highlights that temperature is a key factor regulating NP removal [86].

### 5.4. Complementary Elimination Strategies for Nonylphenol

Studies have shown that adsorption technology can simultaneously reduce the pollution and migration of these substances in the environment [87,88]. Therefore, adsorption has become an effective strategy for the remediation of NP in environmental matrices, serving as a supplement to degradation methods. For example, ferrate (VI) can effectively remove NP from water systems [89]. Research indicates that the adsorption of NP is strongly influenced by the concentration and stability of non-hydrolyzable carbon (NHC) components in the sediment, and adsorption is usually integrated with other removal methods in a mixed system [90]. Experimental results show that hierarchical porous carbon fibers (CFs) provide an energy-saving electrochemical method for NP removal and can synergize with adsorption to enhance this process [91]. A combined adsorption-biodegradation mechanism has also been demonstrated, and decaying leaves colonized by aquatic bacteria can achieve rapid removal of NP from water bodies. In this system, leaves adsorb NP, while bacterial enzymes such as laccase promote its degradation and mineralization [92]. These technologies have been evaluated based on cost, environmental compatibility, degradation rate, and sensitivity to environmental conditions. Although each method has its advantages and disadvantages, they all show the potential for controlling the source of NP.

### 5.5. Environmental Behavior Regulating Factors

Multiple spectroscopic techniques were used to investigate interactions between NP and dissolved organic matter (DOM). These included excitation–emission matrix fluorescence spectroscopy, ultraviolet–visible spectroscopy, Fourier transform infrared spectroscopy, and nuclear magnetic resonance spectroscopy. The results indicated that interactions between NP and DOM are dependent on molecular weight and compositional heterogeneity. This interaction regulates the biological efficacy and environmental mobility of NP [93].

## 6. Toxicokinetic Profiles of Nonylphenol

In toxicological evaluation, physiologically based toxicokinetic (PBTK) data are critical for predicting toxicity and elucidating underlying mechanisms. Contemporary PBTK models enable high-resolution simulation of the absorption, distribution, metabolism, and excretion (ADME) of NP in vivo, with high temporal and spatial precision [94]. Based on data from rats orally administered NP, these models predict serum and tissue concentrations. The results indicate the following rank order of tissue burden: uterus > brain > adipose > ovary > kidney > liver > blood. Quantitative detection of NP in adipose tissue and brain further confirms its ability to cross biological barriers, including the blood–brain barrier.

The high lipophilicity of NP (log Kow = 4.48–4.80) is a key determinant of its tissue distribution and toxicokinetic behavior [41].This physicochemical property facilitates its partitioning into lipid-rich compartments, enabling efficient penetration across biological barriers, including the blood–brain barrier and placental barrier. The ability of NP to accumulate in adipose tissue and the brain has been confirmed by quantitative detection in rodent models, with the brain ranking among the tissues with the highest NP burden following oral administration [95].This preferential accumulation in neural and adipose tissues provides a mechanistic basis for the neurotoxic and endocrine-disrupting effects of NP.

The models also reveal significant entero-systemic shunting. After undergoing metabolism or binding reactions in the intestine, most of the orally administered NP is actively transported into the systemic circulation through the portal vein, while only a small amount of liver metabolites are excreted through bile or venous routes [96]. A detailed study on the liver metabolism of NP demonstrated that NP is conjugated with glucuronic acid through a specific UDP-glucuronosyltransferase subtype UGT2B1 expressed in yeast AH22 cells. This conjugation leads to the retention of free NP and its metabolites in the liver, thereby limiting their excretion via bile or veins. Consistent with this, most NP remained unexcreted in bile even after 1 h of rat liver perfusion [97]. NP also undergoes glucuronidation in the rat intestinal wall; however, direct enteric excretion of NP-glucuronide was not observed [98]. Cytochrome P450 (CYP450) enzymes, predominantly localized in the liver, play a central role in the oxidative metabolism of endogenous compounds and xenobiotics such as NP. Evidence suggests that the cytochrome P450-mediated bioconversion of NP is NADPH-dependent and can be completely inhibited by known P450 inhibitors such as piperonyl butoxide and SKF-525A [99]. Except for rodents, *Rutilus rutilus* was exposed to 4.9 μg/L NP for 4 consecutive days in a flow-through aquarium system. The results showed that the highest residual concentrations and apparent bioconcentration factors (BCF) were found in bile (BCF = 34,121) and liver (BCF = 605), while the BCF values of other tissues ranged from 13 to 250 [100].

In addition, evaluation of NP metabolism in rats of different sexes showed that the clearance of NP from blood was faster in female than in male rats after gavage, and the absorption of NP into blood was faster in male rats. In addition, the distribution and accumulation of NP in all tissues of male rats were higher than those of female rats, suggesting that the sex difference in NP metabolism may result from different absorption processes, clearance processes, or processing differences in different tissues between males and females [101].

## 7. Toxic Effects of Nonylphenol

**Figure 2:**
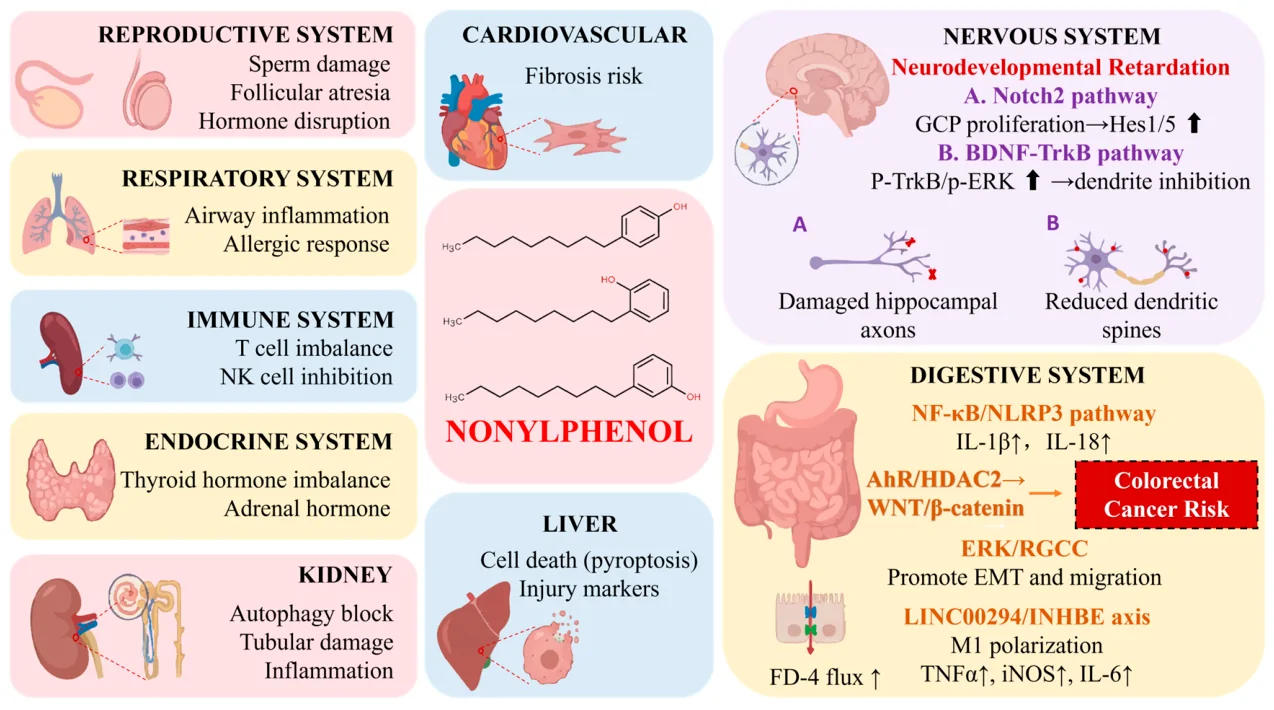
Multi-organ toxic effects induced by nonylphenol.

NP, as a widely existing environmental endocrine disruptor and persistent organic pollutant, can cause multi-organ toxicity, including reproductive [23], nervous [24], digestive [25], endocrine [26], immune [27], respiratory [28], and hepatorenal systems [29] (Figure 2). Its effects are often related to the exposure dose and duration.

### 7.1. Nonylphenol-Induced Neurotoxicity

Based on the toxicological evidence identified through our systematic literature search, NP is prone to accumulate in the central nervous system (CNS) and causes irreversible neurotoxic effects on different developmental stages of mammals [102,103,104]. These effects include neurodevelopmental deficits [105], cognitive impairment [24] (manifesting as anxiety-like and depressive-like behaviors [106,107,108] and memory/learning dysfunction [109]), and potentially neurodegenerative alterations [104]. Accumulating evidence further implicates NP in the pathogenesis of neurodevelopmental disorders, such as ADHD and autism [17,110]. Its neurotoxicity is characterized by impaired synaptic plasticity [111], dysregulated neurotransmitter synthesis and secretion [111,112], and disrupted cerebral homeostasis [109], primarily mediated by inflammatory [111,112] and oxidative stress-related signaling pathways [113]. Experimental studies indicate that perinatal NP exposure induces marked cerebellar hypertrophy in postnatal rats, as evidenced by increased cerebellar weight, surface area, and thickness of the internal granular layer (IGL). This morphological alteration may be attributed to NP-mediated dysregulation of Notch2 signaling, which promotes aberrant proliferation of granule cell precursors (GCPs) within the external granular layer (EGL) of the developing cerebellum [114]. Consistently, perinatal NP exposure suppresses dendritic arborization of cerebellar Purkinje cells in offspring, an irreversible effect mediated by stathmin hyperactivation via the BDNF-TrkB signaling pathway [103]. Developmental NP exposure also compromises axonal integrity, as evidenced by reduced axonal length and density in the developing hippocampus of offspring. These structural defects are associated with the up-regulation of CRMP-2 and phosphorylated CRMP-2 expression and the dysregulation of Wnt-Dvl-GSK-3β signaling cascade [24]. Notably, both axonal malformations and associated signaling imbalances persisted as long-term pathophysiological alterations even after cessation of exposure [24]. Similarly, NP exposure alters transcriptional profiles of key genes in the brains of juvenile Atlantic salmon (*Salmo salar*), affecting expression of genes involved in steroidogenic protein synthesis, xenobiotic metabolism, and cytochrome P450 isoform production [115].

### 7.2. Nonylphenol-Induced Reproductive Toxicity

Based on the evidence synthesized from our systematic literature screening, NP is a persistent organic pollutant and endocrine-disrupting compound present in the environment. Due to its chemical structure being similar to estrogen, the reproductive toxicity induced by the estrogenic effect of NP has always been a research hotspot. The assessment report on gonadotropin-releasing hormone and kisspeptin under NP exposure suggests that NP may interfere with the hypothalamic–pituitary–gonadal axis (HPG axis) through its estrogen-like characteristics, thereby affecting the normal function of the reproductive system [23]. There are two subtypes of mammalian estrogen receptors: ERα and ERβ. Both of these subtypes can initiate transcriptional regulation of downstream target genes by recognizing and binding to estrogen response elements on DNA [116]. In female animals, NP exposure has been proven to weaken the expression of ERβ through its high affinity for ERα, while significantly affecting the transcription of estrogen-responsive genes FSHR and LHCGR, resulting in delayed embryonic development, fetal malformations, and further reduced hatching rates and ovarian dysfunction in offspring [117]. Consistent with this, the reproductive parameters of various fish species, including fertilization rate, fecundity, hatchability, and offspring survival rate, have been significantly inhibited after long-term exposure to NP [118]. In male animals exposed to NP, it can be observed that the integrity of the blood–testis barrier is disrupted [119], the ROS cascade is activated [120], the arrangement of spermatogenic epithelial tissue cells is interfered with [119], and spermatogenesis programs are dysregulated, resulting in increased sperm DNA fragmentation and significant reductions in sperm count, viability, and motility [119,120,121,122]. Through the observation of the toxicological model of NP in medaka (*Oryzias latipes*), researchers have found that the reproductive toxicity of NP may target interstitial homologous cells, Sertoli cells, and spermatogonia rather than directly affecting spermatids. Therefore, the impairment of adult male medaka reproductive capacity caused by brief exposure to NP is not obvious, while long-term exposure to NP significantly induces the loss of testicular cells and degeneration of the testis in medaka [123]. Additionally, there is evidence showing that NP can trigger hormonal imbalance and sex reversal, causing reduced testis size and structural disorders, and even leading to the occurrence of testicular cancer [124].

### 7.3. Nonylphenol-Induced Endocrine-Disrupting Toxicity

The endocrine system maintains organismal homeostasis through tightly regulated hormonal signaling. However, NP, a pervasive endocrine-disrupting chemical, threatens endocrine integrity by interfering with critical biological axes, including the hypothalamic–pituitary axis [110]. Endangered species are particularly vulnerable to environmentally relevant NP concentrations. Endocrine disruption in the Caspian brown trout (*Salmo trutta caspius*) has been observed at a no-observed-effect concentration (NOEC) below 1 μg/L [26]. In a 14-day toxicity assay in juvenile Manila clams (*Ruditapes philippinarum*), NP exposure at 0.1, 1, and 10 μg/L significantly inhibited shell growth. Tissue concentrations of free 17β-estradiol (E2) were markedly elevated on days 7 and day 14. Endocrine responses in juvenile clams exhibited high sensitivity. In females exposed to 1 μg/L NP, mRNA expression of steroidogenesis-related genes, including GnRH, CYP17, and 17β-HSD, was significantly upregulated. Additionally, vitellogenin mRNA expression increased in both sexes [125]. At the hormonal level, mammalian studies further support the endocrine-disrupting effects of NP. Oral administration of NP (5, 25, and 125 mg/kg) to 3-week-old male Wistar rats significantly increased plasma levels of adrenocorticotropic hormones, melatonin, and aldosterone, whereas corticosterone and glucose concentrations remained unchanged [126]. Beyond these hormonal disturbances, NP directly affects the thyroid gland. After 34 weeks of oral exposure to 0.4, 4, and 40 mg/kg NP, results showed dose-dependent accumulation in the thyroid tissue of male rats, and serum FT3 levels were increased in a dose-dependent manner, while FT4 levels showed the opposite trend. These hormonal alterations were associated with increased protein expression of ERα, ERβ, TRα, TRβ, and HMGB1 in thyroid tissue. Moreover, hyperechoic areas (calcifications) were observed in the thyroid of the high-dose group, and a series of pathological changes were observed: disordered follicular epithelial layers, flattened monolayer cuboidal epithelial cells, and significant reduction in the area of individual follicles. The mitochondria of thyroid cells were swollen, and the rough endoplasmic reticulum was proliferated, dilated and vesiculated [127].

### 7.4. Nonylphenol-Induced Hepatorenal Toxicity

Based on the toxicological studies identified through our systematic literature search, the liver, a central organ for metabolism and detoxification, is the primary site of NP biotransformation. Chronic NP exposure induces hepatic dysregulation, manifested as metabolic disturbance, oxidative stress, inflammation, and hepatocyte death. Evidence indicates that NP disrupts hepatic energy-sensing homeostasis, leading to dyslipidemia and chronic inflammation. These effects may involve redox imbalance, upregulation of p38 MAPK signaling, and endoplasmic reticulum stress [29]. Additional studies show that NP-triggered inflammatory cascades activate the pyroptosis hepatocytes. NP-induced DNA methylation–LncRNA interaction regulates the ZBP1 promoter, thereby mediating hepatocyte pyroptosis via PVT1 hypomethylation [128]. In rats, 35 days of gavage administration of NP at 5, 25, and 125 μg/kg elevated serum AST, ALT, and ALP levels and induced histopathological liver damage. This was associated with upregulation of HO-1 and Gadd45b expression [129]. NP-induced nephrotoxicity is primarily characterized by autophagic blockade and pro-inflammatory response. Evidence suggests that graded doses of NP (25, 50, and 75 μL/kg) induce renal tissue deterioration in rats, characterized by tubular epithelial cell degeneration, inflammatory cell infiltration, and hemorrhage. Compared with the controls, kidneys from NP-treated rats showed significantly elevated levels of autophagy markers (Beclin-1, LC3A/B, p62) and pro-inflammatory markers (TNF-α, IL-6, HIF-1α, eNOS). These increases correlated positively with NP dosage. However, only IL-1β upregulation reached statistical significance in NP-exposed renal tissues. These biochemical findings were corroborated by histopathological analysis [130].

Metabolism is the basis for maintaining life activities, and NP exposure has been confirmed to mainly affect lipid metabolism and glucose metabolism. Studies have found that oral exposure to 2.5 mg/kg NP during the critical window of development leads to abnormal metabolic phenotypes in adulthood, as shown by increased body weight and liver fat content in male mice fed a normal fat ratio diet. In contrast, NP exposure altered mRNA levels of the major estrogen receptor alpha (Esr1) and adipose lineage marker genes in both male and female mice fed a high-fat diet. This change showed significant sexual dimorphism and was dependent on the specific fat distribution [131]. Owing to its lipophilic nature, NP accumulates in adipose tissue, thereby contributing to physiological dysregulation [132]. Researchers found that Fabp4 expression was specifically up-regulated by NP in adipocytes derived from primary stromal vascular cells isolated from inguinal white adipose tissue of female mice, but not in males [131]. Furthermore, transcriptomic analyses suggest that NP-mediated inhibition of lipolysis underlies multiple toxic effects in mice, including enhanced lipid storage, impaired growth and reproductive performance, and altered phospholipid composition [132]. This mechanism is further supported by bioinformatic analyses in adult zebrafish. Internal NP exposure correlated with transcriptional changes associated with hepatic steatosis, metabolic disruption, immune dysregulation, and oxidative stress. Collectively, these findings delineate key pathophysiological axes of NP toxicity across vertebrate models [133]. Regarding glucose metabolism, NP enhances glucose uptake and increases the activity of glycolytic enzymes such as hexokinase and pyruvate kinase, in clams, leading to altered glycolytic flux [27].

### 7.5. Nonylphenol-Induced Gastrointestinal System Damage

The gastrointestinal tract is a primary route for systemic absorption of NP. It initiates pathophysiological alterations by dysregulating multiple signaling pathways. Following intraperitoneal administration of ^124^I-radiolabeled NP, rapid biodistribution was observed. Accumulation was detected in the stomach, large intestine, small intestine, and adipose tissue of mice within one hour [134]. In a chronic toxicity study in male SD rats, NP administered at 0.4, 4, and 40 mg/kg induced dose-dependent inflammatory injury in gastric tissues, consistent with the observed distribution pattern. Complementary in vitro experiments using GES-1 gastric epithelial cells exposed to 2.5, 40, and 60 μmol/L NP further demonstrated activation of the NF-κB/NLRP3 signaling pathway in this inflammatory response [25]. Hirschsprung disease typically arises from developmental defects of neural crest-derived cells. However, emerging evidence suggests NP may increase disease susceptibility by promoting M1 macrophage polarization via dysregulation of the LINC00294/INHBE axis [135]. Similarly, NP exposure promotes tumor progression and angiogenesis in rectal cancer by activating the AhR/HDAC2/HNF4α/TL1A signaling cascade [136]. NP suppresses Fyn-related kinase (FRK) expression by upregulating miR-151a-3p. This activates the WNT/β-catenin pathway and promotes cellular proliferation and epithelial–mesenchymal transition (EMT) in rectal carcinoma cells [137]. NP also enhances the invasive and migratory capacities of colorectal cancer cells by reinforcing EMT via activation of ERK signaling and RGCC expression. These findings provide insights into potential therapeutic targets [138]. Experimental studies using the human colon adenocarcinoma Caco-2 cell line showed that transepithelial electrical resistance (TEER) exhibited pronounced time-and dose-dependent sensitivity to NP exposure. Treatment with 10 μM NP rapidly compromised tight junction integrity and paracellular permeability in Caco-2 monolayers. Detectable alterations occurred within 15 min. After six days of exposure to 1 or 10 μM NP, galectin-3, GSTA2, and peroxiredoxin-1 (Prx-1) were coordinately upregulated. This suggests a protective adaptive response to NP-induced cellular stress [139].

### 7.6. Nonylphenol-Induced Respiratory Impairment

To date, research on the direct inhalational toxicity of NP to the respiratory system remains limited. However, NP can induce respiratory injury through multiple exposure routes. Studies show NP significantly exacerbates ovalbumin-induced inflammatory responses in allergic rhinitis by activating the TSLP-TSLPR/IL-7R and JAK1/2-STAT3 signaling pathways [28]. Marked histopathological alterations in fish gill microstructure have been used as reliable indicators of NP contamination in aquatic environments. These findings provide a basis for its regulation and safe disposal [140]. Low-dose prenatal exposure to NP is recognized as a risk factor for childhood asthma. This finding is supported by gestational rat toxicity models. Following daily intragastric administration of NP (25, 50, and 100 mg/kg) from gestational day 7 to postnatal day 21, dose-dependent accumulation of NP was detected in lung tissues of offspring. This was accompanied by dose-dependent upregulation of HMGB1, NF-κB, and ER-β at both the mRNA and protein levels. In the high-dose group, histopathological examination revealed significant abnormalities. Bronchiolar smooth muscle was markedly thickened. Extensive vacuolization was observed in osmiophilic lamellar bodies of type II epithelial cells. Additionally, lymphocyte and neutrophil counts were significantly elevated in bronchoalveolar lavage fluid (BALF). These findings indicate that perinatal NP exposure has multiple effects on offspring. It activates inflammatory pathways and induces inflammatory responses. It also promotes the infiltration of inflammatory cells into the bronchi and bronchioles. Furthermore, it induces significant pathological remodeling of airway structures [141]. Chronic hypoxia and inflammation resulting from NP-induced respiratory toxicity may trigger myocardial fibrosis. Studies indicate that NP activates the RhoA/ROCK1/MLC signaling pathway, leading to cardiac fibroblast activation and proliferation, and contributing to myocardial fibrosis. However, the direct link between this cardiac effect and NP-driven respiratory toxicity requires further experimental validation [142].

### 7.7. Nonylphenol-Induced Immunotoxicity

Accumulating evidence indicates that NP exposure disrupts immune responses and immunological function in both animals and humans. NP exposure activates both the ERK-NOX-ROS signaling axis and the glycolytic pathway, and this dual activation promotes the formation of extracellular vesicles, leading to cellular immune dysfunction in Manila clam (*Ruditapes philippinarum*) [27]. Similarly, NP contamination disrupts the immune defenses of other marine bivalve mollusks. In scallops, this manifests as altered transcription of immune-related genes, and antioxidant enzyme activity is also significantly inhibited in tissues [143]. Direct experimental evidence further supports the developmental and humoral immunotoxicity of NP. In a study, pregnant rats (F0 generation) were continuously exposed to low-dose NP from day 6 of pregnancy (GD6) to 90 days post-natal (PND90), a period covering the critical period of immune system maturation; their F1 offspring continued to be exposed until PND42. Comprehensive immunological evaluation showed significant sexual dimorphism in the immunotoxicity induced by NP exposure in early life, with female offspring being more sensitive. These immunotoxic effects were characterized by reduced thymus and spleen weights, suppressed humoral immunity, decreased NK cell activity, and an imbalanced Th17/Treg cell ratio. In contrast, male offspring showed only reduced NK cell activity, with no significant changes in humoral immune function [144].

### 7.8. Combined Toxicity of Nonylphenol

Environmental contaminants rarely occur in isolation under realistic conditions. Accordingly, NP produces distinct toxicological outcomes through interactions with other co-existing environmental factors. Biomarker analysis in juvenile carp exposed to polyethylene microplastics (PE-MPs) and NP revealed dose-dependent damage that was more severe than that caused by either contaminant alone, indicating a synergistic effect of combined exposure [33]. Consistently, Caco-2 cells co-exposed to PE-MPs and NP exhibited intensified oxidative stress and inflammatory injury. This effect was mediated by hyperactivation of MAPK signaling, leading to cell proliferation arrest and apoptosis [145]. Conversely, a comparative analysis of the individual and combined toxicity of NP and microplastics (polyethylene [PE], polystyrene [PS], and polyamide [PA]) in the alga Chlorella pyrenoidosa demonstrated an antagonistic interaction. Although NP and microplastics alone significantly inhibited algal growth, their combination resulted in reduced inhibition. This was accompanied by changes in chlorophyll fluorescence, malondialdehyde (MDA) levels, and the activities of antioxidant enzymes, including superoxide dismutase (SOD) and catalase (CAT) [146]. In addition, organisms may be exposed to both NP and heavy metals in the environment. In zebrafish liver, co-exposure to environmentally relevant concentrations of NP (45.38 nM, 453.8 nM) and cadmium (Cd, 54.55 nM) exacerbated ROS accumulation, MDA production, and DNA damage compared with single exposures. This synergistic interaction further amplified hepatic enzyme inactivation, inflammatory cytokine secretion, microstructural tissue damage, and abnormal gene transcription induced by individual pollutants [147]. Beyond microplastics and metals, combined administration of genistein and NP aggravates lung carcinogenesis in rats, involving oxyradical-mediated proliferation arrest and DNA damage [148]. Murine sertoli cells also exhibited enhanced toxic injury following co-exposure to pyrethroids and NP [149]. Beyond individual pollutant classes, studies on mixtures of endocrine-disrupting chemicals indicate that NP and octylphenol alter the expression of serotonergic components via activating estrogen receptors (ER). The affected components included the levels of tryptophan hydroxylase 2 (TPH2), monoamine oxidase A (MAOA), serotonin transporter (SERT) and vesicular monoamine transporter 2 (VMAT2), which affected the content of 5-hydroxytryptamine (5-HT) in PC12 cells and rat neurons. It induces disruption of the cholinergic system and impaired learning and memory abilities [150,151]. A summary of the mixed toxicity of NP and other pollutants is shown in Table 2.

## 8. Advances in the Toxic Mechanisms of Nonylphenol

### 8.1. Nonylphenol-Induced Oxidative Stress

Up to now, the researchers’ exploration of the core toxicity mechanism of NP, focusing on oxidative stress, has reached a mature stage. It has been confirmed that NP in the environment can exacerbate the destruction of the antioxidant defense mechanism in juvenile tilapia (*Oreochromis niloticus*) induced by heat stress, further promoting the accumulation of ROS [16]. An increasing number of investigations have confirmed that not only in aquatic organisms, but also in different systems and tissues of mammals, the activities of antioxidant markers such as SOD, CAT, glutathione peroxidase (GPx), and glutathione reductase (GR) usually decrease due to the stimulation of NP, accompanied by an increase in ROS and MDA levels [108,113,152,153,154,155]. It has been confirmed that after continuous intragastric administration of NP at doses of 1, 10, and 100 μg/kg for 45 days to male rats, the levels of sperm lipid peroxidation (expressed as the equivalent of H_2_O_2_ per milligram of protein and per milligram of DNA) show a dose-dependent significant increase, indicating that the antioxidant defense system in sperm is depleted due to NP, thereby significantly promoting the oxidative stress of sperms in the epididymis of rats [152]. In the central nervous system, oxidative stress-induced endoplasmic reticulum stress may lead to a reduction in dendritic spines and synaptic dysfunction, which is considered to be the potential basis for neurobehavioral abnormalities in mammals exposed to NP in early life [17].

### 8.2. Nonylphenol-Induced Mitochondrial Injury

In veterinary toxicology studies, the interaction between ROS and mitochondria has been identified as a key initiating event in NP-induced mitochondrial dysfunction and structural damage. Indirect and direct evidence were obtained in the mouse oocyte model exposed to NP. JC-1 staining and fluorescence intensity quantification showed that elevated ROS levels coincided with a significant decrease in mitochondrial membrane potential (MMP) [15]. Transmission electron microscopy (TEM) confirmed the abnormal mitochondrial localization induced by NP, providing direct evidence of mitochondrial destruction. In addition, immunofluorescence imaging revealed meiotic spindle disorder and chromosome misalignment in oocytes. These findings indicate that NP exposure induces mitochondrial dysfunction. Further analysis revealed early apoptotic signals in NP-treated oocytes. These findings suggest that NP compromises oocyte quality by disrupting cytoskeletal dynamics and mitochondrial integrity, ultimately triggering apoptotic pathways [156]. Furthermore, perinatal NP exposure in rats induced dose-dependent mitochondrial impairment in offspring. In vitro cellular assays further suggest that NP-induced mitochondrial perturbations may result from suppression of the PGC-1α/NRF-1/TFAM signaling pathway. These findings are derived from veterinary toxicological studies [157].

### 8.3. Nonylphenol-Induced Perturbations of the Endoplasmic Reticulum and Golgi

The endoplasmic reticulum and Golgi apparatus are essential for maintaining cellular structure and functional integrity. Their pathophysiological responses to NP exposure have been increasingly characterized in veterinary toxicology. Studies indicate that NP induces endoplasmic reticulum stress, primarily mediated by ROS. This process contributes to dendritic spines loss and synaptic dysfunction in the central nervous system [17]. Following high-dose exposure to NP, a disrupted endoplasmic reticulum network was observed in the ooplasm of murine oocytes. This network exhibited irregular and aggregated structures. Furthermore, molecular analyses using Western blot and RT-PCR showed that NP exposure significantly increased mRNA and protein levels of ER stress markers GRP78 and ATF4 in oocytes. Immunofluorescence intensity quantification provided additional evidence of ER stress induction [15].

As a key organelle in the cellular secretory machinery, the Golgi apparatus is highly susceptible to functional impairment following structural disruption. Vesiculation of the Golgi apparatus is considered a hallmark of NP-induced cellular perturbation. A gradient of sensitivity is observed across different experimental models. Epithelial papulosum cyprini cell lines show the highest susceptibility, followed by primary epithelial cells, whereas explant tissues are the least responsive. Notably, Golgi vesiculation and degenerative restructuring were observed only in the two cultured cell lineages [18].

### 8.4. Nonylphenol-Induced Programmed Cell Death

**Figure 3:**
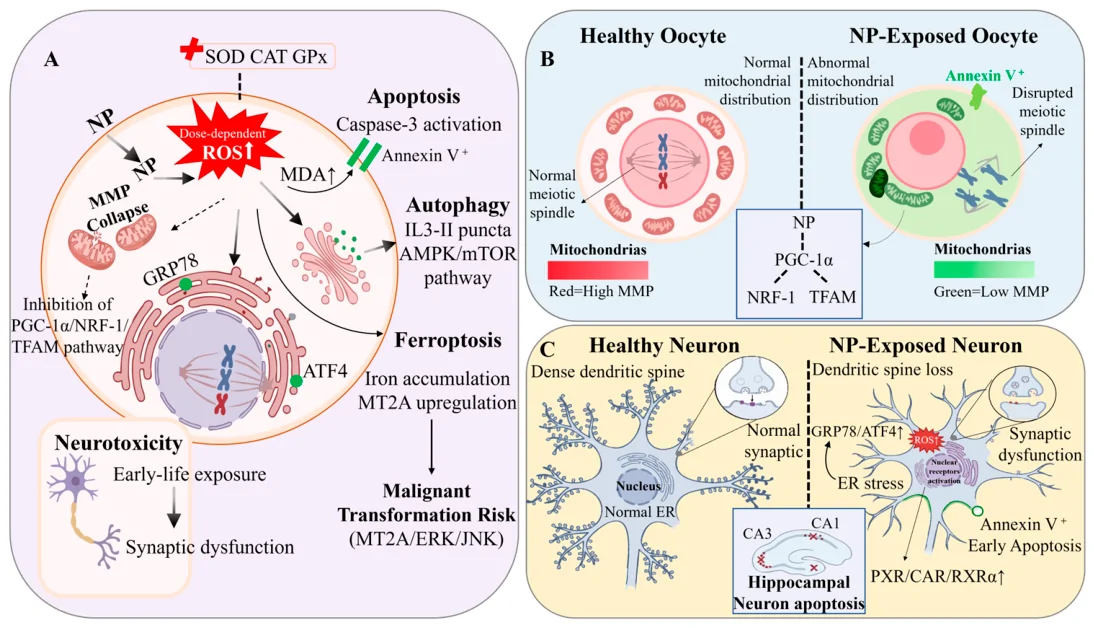
Molecular mechanism network of nonylphenol toxicity. (**A**): NP-induced comprehensive cell damage. (**B**): NP-induced oocyte deterioration. (**C**): Signaling pathways and cell death crosstalk in NP toxicity.

NP induces multiple forms of programmed cell death, including apoptosis, autophagy, and ferroptosis. Early evidence indicates that these mechanisms are primarily regulated by ROS homeostasis, caspase activation, and MMP disruption. In *Litopenaeus vannamei*, NP induces time- and concentration-dependent programmed cell death [158]. In addition, studies indicate that ROS-mediated activation of AMPK/Akt/mTOR and JNK pathways contributes to NP-induced autophagy and apoptosis in Sertoli cells and granulosa cells (GCs) [20]. NP disrupts aerobic glycolysis by downregulating HIF-1α, a key regulator of this pathway. Conversely, enhancement glycolysis mitigates NP-induced ROS generation and reduces apoptosis in Sertoli cells [159]. Similarly, A Disintegrin and Metalloproteinase 17 (ADAM17) may alleviate NP-induced oxidative stress and apoptosis [160]. In addition to ROS, caspases and MMP play key roles in NP-induced extrinsic and intrinsic apoptotic pathways [161,162]. Inhibition of caspase-8 with IETD-fmk at 12 and 24 h post-exposure prevented MMP loss and reversed apoptosis in Jurkat cells treated with NP (0.1, 1, and 10 μM). Similarly, targeting caspase-3 reduced NP-induced apoptosis by modulating mitochondrial depolarization [161]. Furthermore, siRNA and immunofluorescence analyses revealed a key mechanism. NP exposure upregulates retinoid X receptor, pregnane X receptor, and constitutive androstane receptor at both the mRNA and protein levels. This upregulation contributes to increased caspase-3 and lactate dehydrogenase levels. These changes ultimately induce apoptosis in mouse primary neurons and hippocampal cells [21]. In PC12 cells, serum withdrawal-induced apoptosis is associated with increased caspase-3 activity [163]. Among non-apoptotic forms of cell death, ferroptosis is an iron-dependent process that contributes to NP-induced cellular toxicity. In a contrasting adaptive response, 30 weeks of NP exposure in normal human cervical epithelial cells upregulated MT2A and increased ERK1/2 and JNK/MAPK phosphorylation. This response enhances antioxidant capacity and promotes clearance of toxic iron ions. This alteration in ferroptosis may contribute to NP-mediated neoplastic transformation of cervical epithelial cells, although further investigation is required [22]. Whole-genome transcriptomic profiling in *S. cerevisiae* BY4742 exposed to 2 mg/L NP identified 948 differentially expressed genes (834 downregulated, 114 upregulated). Functional enrichment analysis further implicated these genes in cell cycle regulation, autophagy, ferroptosis, and copper uptake. Together, these findings provide a transcriptional framework for NP-induced fungal responses, particularly in relation to programmed cell death [164] (Figure 3).

## 9. Regulation of Absorption and Promotion of Excretion

Source reduction, including regulatory restrictions, clean production, and wastewater treatment, remains the primary approach to minimizing NP exposure. Exploring auxiliary mitigation strategies at the biological level is a complementary avenue in toxicological research. The strategies discussed below are primarily experimental and validated only in laboratory models. They are not intended as substitutes for regulatory control or pollution prevention. They serve as tools for mechanistic validation.

### 9.1. Regulation Strategies of Nonylphenol Absorption, Efflux and Degradation

Bifidobacterium longum CCFM1077 is a probiotic strain with strong in vitro NP-binding capacity. In rats, it mitigates NP-induced toxicity by promoting NP excretion. This enhances intestinal barrier integrity, upregulates antioxidant-related metabolic pathways, reduces cerebral inflammation, and alleviates behavioral deficits [165].

### 9.2. Strategic Orchestration of Antioxidant, Anti-Inflammatory, and Anti-Cell Death Pathways

(i) Selenium (Se): The question of whether Se can alleviate NP-induced testicular toxicity in rats has been answered. The oxidative stress induced by NP was significantly inhibited by Se, as judged by cytological, histological, biochemical and immunohistochemical methods, accompanied by the recovery of MDA and SOD levels in serum and testis. Compared with the NP group, the renal tubular density was significantly increased, Caspase-3 was significantly decreased, and the number of viable and normal sperm was significantly increased in the Se group [166].

(ii) RES and NG: RES and NG are regarded as active compounds derived from natural plants with antioxidant properties and estrogenic activity. In the toxicological model of male rats, all the toxic effects induced by NP administration could be effectively counteracted by the protective effects of RES or NG [34].

(iii) Tea polyphenols (TPs) and tea polysaccharides (TPSs): TPs and TPSs were found to attenuate NP-induced neuroinflammation and depression-like behaviors in rodents by targeting inhibition of neuronal apoptosis and blocking monoamine neurotransmitter secretion disruption [167].

(iv) Thymoquinone (TQ): As a major bioactive compound in the seed of Nigella sativa, TQ has been found to have antioxidant and anti-inflammatory properties. Based on this, TQ can effectively protect rats against NP-induced neurotoxicity and memory deficits [168].

(v) Aucubin (AU): AU, a potential antioxidant and anti-apoptotic natural compound in the iridoid class, attenuated testicular cell apoptosis by inhibiting the expression of Bax, Caspase-9 and Caspase-3 in the NP exposure experiment. This was accompanied by alleviation of testicular histopathological damage, characterized by the recovery of seminiferous tubule diameter and the height of the epithelium and lamina propria. Moreover, the abnormality (head/midpiece/tail) and death of sperm induced by NP were also significantly alleviated by AU [169].

(vi) Melatonin (MT): MT can prevent the in vitro cytotoxicity induced by NP through antioxidation and anti-DNA damage [170,171,172]. The mitochondrial dysfunction and ROS accumulation in INS-1 cells are ameliorated due to the MT-mediated MDM2-P53-P21 axis [172]. In vivo, MT maintains cellular proteostasis through two mechanisms: restoration of RPS3 expression to preserve ribosomal function, and suppression of GRP78 to reduce endoplasmic reticulum stress and associated morphological alterations. In mice exposed to 500 μg/L NP, administration of melatonin (30 mg/kg) prevented mitochondrial and metabolic dysfunctions, including abnormal mitochondrial distribution, loss of MMP, impaired DNA replication, and ATP depletion [170]. The protective effects of MT were further confirmed in a complementary in vivo model. Co-administration of MT (10 mg/kg) with NP (25 mg/kg) for 45 days mitigated systemic toxicity in rats. This effect was associated with improved neurobehavioral outcomes. Improvements were observed in maze navigation, grip strength, and rotarod performance [171].

(vii) Yam polysaccharides (YP): A seleno-functionalized YP, synthesized via covalent selenite grafting, protects against NP-induced enterocyte toxicity and intestinal barrier dysfunction in rats, primarily by inhibiting MAPK signaling [173].

(viii) Boric acid: Boric acid protects against NP-induced ovarian injury in female rats. This protection is associated with the restoration of serum anti-Müllerian hormone and stem cell factor levels. Additionally, boric acid restores ovarian SOD and CAT levels. It also reduces DNA damage and mitigates the levels of proinflammatory IL-6 and TNF-α. These effects are mediated by upregulation of SIRT1 and mTOR [174].

While these findings collectively demonstrate the potential of various natural compounds and trace elements to counteract NP-induced toxicity in experimental settings, several critical limitations must be acknowledged. First, the majority of these studies employed acute or subacute exposure models with NP concentrations far exceeding environmentally relevant levels, leaving the efficacy of these protective agents under chronic low-dose exposure scenarios largely unknown. Second, the administration protocols—including timing, dosage, and route of delivery—varied considerably across studies, making direct comparisons and identification of optimal intervention strategies difficult. Third, the safety profiles and potential side effects of these compounds themselves, particularly at the doses required to achieve protection, have not been systematically evaluated. Therefore, while these findings provide valuable mechanistic insights into NP toxicity and suggest potential adjunct strategies for high-risk exposure scenarios, they should not be interpreted as immediate public health interventions. Future research should prioritize dose–response optimization, long-term safety assessment, and validation in more environmentally relevant exposure models before any translational application can be considered.

### 9.3. Strategic Orchestration of Endocrine and Bio-Signaling Homeostasis

(i) Estrogen receptor β (ERβ): ERβ, which activates the TPH2/5-HT signaling pathway, may mitigate NP-induced depression-like behaviors, serum hormonal disturbances, and impaired monoamine neurotransmitter secretion [175].

(ii) Lycium Barbarum Polysaccharides (LBPs): NP induces neurodevelopmental deficits via the p38/5-HT1A/Wnt/β-catenin signaling cascade, which is inhibited by LBP. This neuroprotection effect is attributed to activation of p38-mediated SIRT1/MAOA and CREB/BDNF/TrkB signaling pathways [151,176]. A summary of intervention strategies and protective agents against nonylphenol toxicity is shown in Table 3.

## 10. Conclusions and Future Perspective

Nonylphenol (NP) is a representative environmental contaminant with both endocrine-disrupting and persistent organic pollutant properties. This review demonstrates that NP poses widespread and multifaceted risks to ecosystems, wildlife, and human health. NP, a degradation product of NP ethoxylates, is widely distributed across environmental compartments, including air, water, and soil. Its environmental recalcitrance, high hydrophobicity, and bioaccumulative properties drive trophic chain magnification, creating exposure pathways from the environment to wildlife, livestock, and ultimately humans. NP induces systemic toxicity affecting the reproductive, neurological, gastrointestinal, hepatic, renal, respiratory, and immune systems. These mechanistic pathways are intricately interconnected. Oxidative stress and mitochondrial dysfunction act as key initiating events, leading to endoplasmic reticulum stress and Golgi apparatus disruption, and ultimately resulting in programmed cell death including apoptosis, autophagy, and ferroptosis. The health risks of NP are characterized by multisystem involvement and interconnected mechanisms, as illustrated in Figure 2 and Figure 3. NP also exhibits significant combined toxicity under environmentally realistic conditions. Co-exposure with microplastics, heavy metals, and other co-occurring pollutants frequently elicits synergistic toxicity, further amplifying its ecological risk.

However, significant deficiencies persist in current research. Industrial-grade NP is a complex mixture of over a hundred isomers, yet existing studies mostly focus on overall toxicity, lacking systematic exploration of isomer-specific differences. Combined toxicity studies are largely limited to binary pollutant mixtures, while actual environmental systems contain multiple pollutants. Remediation technologies are mostly validated under laboratory conditions, with limited field application. Finally, health effect studies rely heavily on animal models, lacking long-term human cohort data, leaving dose–response relationships and sensitive population screening unclear.

This review has several methodological limitations. First, we only retrieved literature from PubMed and Web of Science databases and included only English-language publications, which may have resulted in the omission of relevant studies published in other languages. Second, the included studies varied considerably in exposure protocols (dose, duration, and route), experimental models, and measured endpoints, making it difficult to perform a quantitative meta-analysis. Consequently, our qualitative synthesis may carry a degree of selective reporting bias. Third, the current evidence on NP’s health effects is derived predominantly from animal experiments and in vitro studies, with limited epidemiological data from human cohorts. Extrapolating these laboratory findings to real-world human health risks therefore entails considerable uncertainty. These limitations highlight the need for standardized toxicological study designs and long-term population-based monitoring to refine the risk assessment framework for NP.

Future research should focus on four key directions. First, isomer-specific toxicology studies are critically needed to clarify the environmental behavior and toxicological characteristics of different NP isomers, and to establish refined risk assessment models for regulatory decision-making. Second, research on combined toxicity in complex environmental matrices should be deepened to explore interactions between NP and co-occurring pollutants such as microplastics, heavy metals, and pesticides, and to develop multi-pollutant joint risk assessment systems. Third, remediation technologies require enhanced field-scale validation, optimization of microbial degradation and advanced oxidation processes under site-specific conditions, and the development of integrated approaches such as adsorption-biodegradation and nano-phyto-microbial collaboration. Fourth, long-term human biomonitoring and cohort studies are essential to identify susceptible populations, clarify dose–response relationships, and establish health protection standards and targeted intervention strategies.

In conclusion, NP, as a persistent environmental toxicant, has been widely confirmed to have environmental hazards and biological toxicity effects. Research on the related toxicological mechanisms and risk mitigation strategies has also made certain progress. However, the current studies still have shortcomings in terms of isomer specificity, combined toxicity, technology transfer, and human health research. Future research needs to break through disciplinary boundaries and conduct in-depth and systematic investigations in the above key directions to build a new risk management system for NP. This is not only of great significance for maintaining ecological biodiversity and animal welfare, but also a core guarantee for ensuring global public health security and maintaining the stability of the Earth’s ecosystem.

## Figures and Tables

**Table 1:** Regulatory standards and measures for nonylphenol (NP) in major countries and regions.

Country/Region	Regulatory Measures/Limits	Basis/Remarks	Reference
European Union (EU)	1. NP and NPEs shall not be placed on the market or used as substances or in mixtures at concentrations ≥ 0.1% by weight for industrial and institutional cleaning, domestic cleaning, textiles and leather processing, agricultural teat dips, metal working, pulp and paper manufacturing, cosmetics, personal care products (except spermicides), and as co-formulants in pesticides and biocides; 2. NP is classified under CLP as Repr. 2. H361fd—Suspected of damaging fertility. Suspected of damaging the unborn child.	1. REACH Regulation Annex XVII, Entry 46; 2. CLP Regulation (EC No 1272/2008) Annex VI	[55,56]
United States (Minnesota)	Minnesota sets a maximum concentration of 20 μg/L	MDH Drinking Water Health Guidance Value	[57]
Japan	NP is listed as a Hazardous Air Pollutant under the Air Pollution Control Act.	Cabinet Order No. 165, Appendix 1 of the 9th Report of the Central Environment Council	[58]
China	Risk management measures for NP are primarily implemented through source-based use restrictions, including bans on the use of NP as an additive in pesticide production, as an ingredient in cosmetics, and in the manufacture of nonylphenol ethoxylates (NPEOs).	Priority Control List of New Pollutants (2023 Edition)	[59]

**Table 2:** Mixed toxicity profile.

Co-Occurring Ecological Determinants	Toxicological Paradigm	Toxicological Outcomes	Interactive Modality	Reference
PE-MPs	*Cyprinus carpio* juveniles	Oxidative insult; Inflammatory perturbation	Synergistic effect	[33]
	Caco-2 cells	Proliferative blockade; Apoptosis	Synergistic effect	[145]
PE, PS, PA	*Chlorella pyrenoidosa*	Growth inhibition; Alterations in antioxidant enzymatic repertoire	Antagonistic effect	[146]
Cd	Zebrafish liver	ROS accumulation; DNA strand breaks; MDA generation	Synergistic effect	[147]
Genistein	Rat lung	Proliferative blockade; DNA strand breaks	Synergistic effect	[148]
Pyrethroid	Murine Sertoli cells	Cytopathic effect	Synergistic effect	[149]
Octylphenol	PC12 cells, Rat neural systems	Disturbance in 5-hydroxytryptamine (5-HT) dynamics; Compromised cognitive performance	Synergistic effect	[150,151]

**Table 3:** Intervention strategies and protective agents against nonylphenol toxicity.

Protective Agent	Mechanism of Action	Key Effects	Reference
Bifidobacterium longum CCFM1077	Facilitates NP excretion; enhances intestinal barrier integrity; upregulates antioxidant-associated pathways	Mitigates NP-induced toxicity; ameliorates cerebral inflammation and behavioral deficits	[165]
Se	Suppresses NP-induced oxidative stress; abrogates Caspase-3 activation; normalizes seminal oxidative stress markers (MDA, SOD)	Alleviates testicular toxicity; elevates renal tubule density; upregulates populations of motile and morphologically intact spermatozoa	[166]
RES and NG	Exhibits antioxidant capabilities and estrogenic signaling potential	Counteracts the full spectrum of NP-induced toxic effects in a male rat model	[34]
TPs and TPSs	Suppresses neuronal apoptosis; abrogates monoamine neurotransmitter secretion disruption	Mitigates neuroinflammation and depression-like behaviors in rodents	[167]
TQ	Exhibits antioxidant and anti-neuroinflammatory capacities	Mitigates neurotoxic pathology and associated memory deficits in rats	[168]
AU	Suppresses expression of apoptotic mediators Bax, Caspase-9, and Caspase-3	Facilitates restoration of testicular architecture; ameliorates sperm anomalies and reduces sperm mortality	[169]
MT	Antioxidant and anti-DNA damage; modulates MDM2-p53-p21 axis; reinstates RPS3 expression and suppresses GRP78	Confers cytoprotection in vitro; averts mitochondrial/metabolic dysfunction and neurobehavioral impairments in vivo	[170,171,172]
YP	Suppresses MAPK signaling transduction	Protects against enterocyte toxicity and impairment of the intestinal barrier in rats	[173]
Boric Acid	Upregulates SIRT1 and downregulates mTOR; attenuates DNA damage and proinflammatory cytokines	Confers protection against ovarian injury; restores serum AMH and SCF levels	[174]
ERβ	Activates the TPH2/5-HT signaling pathway	Mitigates depression-like behaviors, serum hormonal disturbances, and impairments in monoamine secretion	[175]
LBP	Engages p38-mediated SIRT1/MAOA and CREB/BDNF/TrkB signaling axes; abrogates p38/5-HT1A/Wnt/β-catenin cascade	Confers neuroprotection against neurodevelopmental deficits	[151,176]

## Data Availability

The data presented in this study are available in publicly accessible databases. The bibliographic references analyzed for the literature review were derived from the PubMed database (https://pubmed.ncbi.nlm.nih.gov/ (accessed on 31 January 2026)).

## Abbreviations

The following abbreviations are used in this manuscript:

NP	Nonylphenol
NPEO	Nonylphenol polyoxyethylene ethers
NPEs	Nonylphenol ethoxylates
MEC	Microbial electrolysis cell
MIP–GC–MS	Molecularly imprinted polymer-assisted gas chromatography–mass spectrometry
RES	Resveratrol
NG	Naringenin
NPnEOs	Nonylphenol polyethoxylates
NP2EO	Nonylphenol diethoxylate
NP1EO	Nonylphenol monoethoxylate
NP2EC	Nonylphenoxyethoxyacetic acid
NP1EC	Nonylphenoxyacetic acid
HPG axis	Hypothalamic–pituitary–gonadal axis
CNS	Central nervous system
GCPs	Granule cell precursors
EGL	External granular layer
FRK	Fyn-related kinase
EMT	Epithelial–mesenchymal transition
TEER	Transepithelial electrical resistance
Prx-1	Peroxiredoxin-1
BALF	Bronchoalveolar lavage fluid
PBTK	Physiologically based toxicokinetic
ADME	Absorption, distribution, metabolism, and excretion
CYP450	Cytochrome P450
BCFs	Bioconcentration factors
Esr1	Estrogen receptor alpha
PE-MPs	Polyethylene microplastics
PE	Polyethylene
PS	Polystyrene
PA	Polyamide
MDA	Malondialdehyde
SOD	Superoxide dismutase
CAT	Catalase
Cd	Cadmium
ERs	Estrogen receptors
TPH2	Tryptophan hydroxylase 2
MAOA	Monoamine oxidase A
SERT	Serotonin transporter
VMAT2	Vesicular monoamine transporter 2
5-HT	5-hydroxytryptamine
GPx	Glutathione peroxidase
GR	Glutathione reductase
MMP	Mitochondrial membrane potential
TEM	Transmission electron microscopy
GCs	Granulosa cells
ADAM17	A Disintegrin and Metalloproteinase 17
Se	Selenium
DOM	Dissolved organic matter
TPs	Tea polyphenols
TPSs	Tea polysaccharides
TQ	Thymoquinone
AU	Aucubin
MT	Melatonin
YPs	Yam polysaccharides
ERβ	Estrogen receptor β
LBPs	Lycium Barbarum Polysaccharides
PLE	Pressurized liquid extraction
GC-MS	Gas chromatography–mass spectrometry
SPE	Solid-phase extraction
UPLC-MS/MS	Ultra-high performance liquid chromatography–tandem mass spectrometry
HPLC–ESI–MS	High-performance liquid chromatography coupled with electrospray ionization mass spectrometry
MAE	Microwave-assisted extraction
TCS	Triclosan
AuNPs	Gold nanoparticles
HRP	Horseradish peroxidase
WRF	White-rot fungi
NHC	Non-hydrolyzable carbon
CFs	Carbon fibers
